# Giant Benign Prostatic Hyperplasia: A Case Report

**DOI:** 10.7759/cureus.61295

**Published:** 2024-05-29

**Authors:** Marika Mdivnishvili, Nutsa Khuskivadze, Alexandre Khuskivadze

**Affiliations:** 1 Cardiac, Thoracic, and Vascular Surgery, Jerarsi Hospital, Tbilisi, GEO; 2 Cardiology, Tbilisi State Medical University, Tbilisi, GEO; 3 Urology, Jerarsi Clinic, Tbilisi, GEO

**Keywords:** lower urinary tract symptoms, giant benign prostatic hyperplasia, transvertical prostatectomy, giant prostatic hyperplasia, benign prostatic hyperplasia

## Abstract

We present the case of a 69-year-old man experiencing lower urinary tract symptoms (LUTS), notably difficulties with urination. His total prostate-specific antigen level was measured at 3.52 ng/ml, accompanied by an International Prostate Symptom Score of 32. Transrectal ultrasound revealed a prostate volume of 268 cm^3^. Benign prostatic hyperplasia (BPH) is a common condition among aging men, often manifesting as LUTS. However, in rare instances, BPH can progress pathologically to giant prostatic hyperplasia, characterized by a prostate gland exceeding 500 g in weight. This report documents the successful enucleation of the giant BPH without significant complications, utilizing a transvesical prostatectomy technique. Our case underscores the importance of early diagnosis and appropriate management strategies.

## Introduction

Approximately one-third of men aged over 60 experience enlargement of the prostate gland due to benign prostatic hyperplasia (BPH), which can cause worsening urinary tract symptoms. Occasionally, the prostate can enlarge significantly, surpassing 500 g in weight, a condition termed giant prostatic hyperplasia (GPH). BPH is detectable in 30-40% of men by their fourth decade of life, with its prevalence increasing almost linearly to 70-80% in those over 80 years old [1,2]. While the pathophysiology of BPH/benign prostatic enlargement (BPE) is not fully understood, the androgen system and androgen receptors play a significant role. Prostatic hyperplasia occurs due to the proliferation of epithelial and stromal cells, impaired apoptosis, and endocrine control. As men age, an enlarged prostate typically leads to the storage and voiding of lower urinary tract symptoms (LUTS), although there is not a direct correlation between prostate size and symptom severity [3]. Despite the prevalence, extremely large prostates, particularly those exceeding more than 500 ml without malignant components, remain rare. No more than 10 cases of GHP reaching 700 mL have been reported in the medical literature [4,5]. In this case, a patient underwent successful removal of a massively enlarged prostate (600 g) via transvesical prostatectomy. The prostate adenoma was removed through an intravesical approach, and an epicystostomy tube along with a urethral balloon catheter was inserted into the bladder.

## Case presentation

A 69-year-old male was admitted to the hospital with a history of worsening LUTS. He was experiencing irritative, obstructive, and voiding symptoms, which culminated in acute urinary retention and significantly impacted his quality of life. His International Prostate Symptom Score (IPSS) was notably elevated at 32, indicating severe impairment in urinary function. There was no history of hematuria or carcinogen exposure. The urine culture showed no bacterial growth. Ultrasonography of the urinary tract revealed bilateral kidney dilation (22 mm on the left and 27 mm on the right), and additionally, bladder ultrasound demonstrated a capacity of 980 ml with a significant residual urine volume of 450 ml. A digital rectal examination revealed a markedly enlarged prostate gland, which was protruding markedly, and a calculated volume of 268 cm3. Laboratory investigations showed a prostate-specific antigen level of 3.52 ng/ml and a serum creatinine level of 104 mmol/l. The maximum urine flow rate (Qmax) was measured at 7 ml/s, indicating obstructive uropathy.

During the patient’s consultation, potential complications of the procedure were thoroughly discussed, including retrograde ejaculation and attendant fertility challenges. Treatment options and better alternative techniques were explained as potentially superior options.

**Figure 1:**
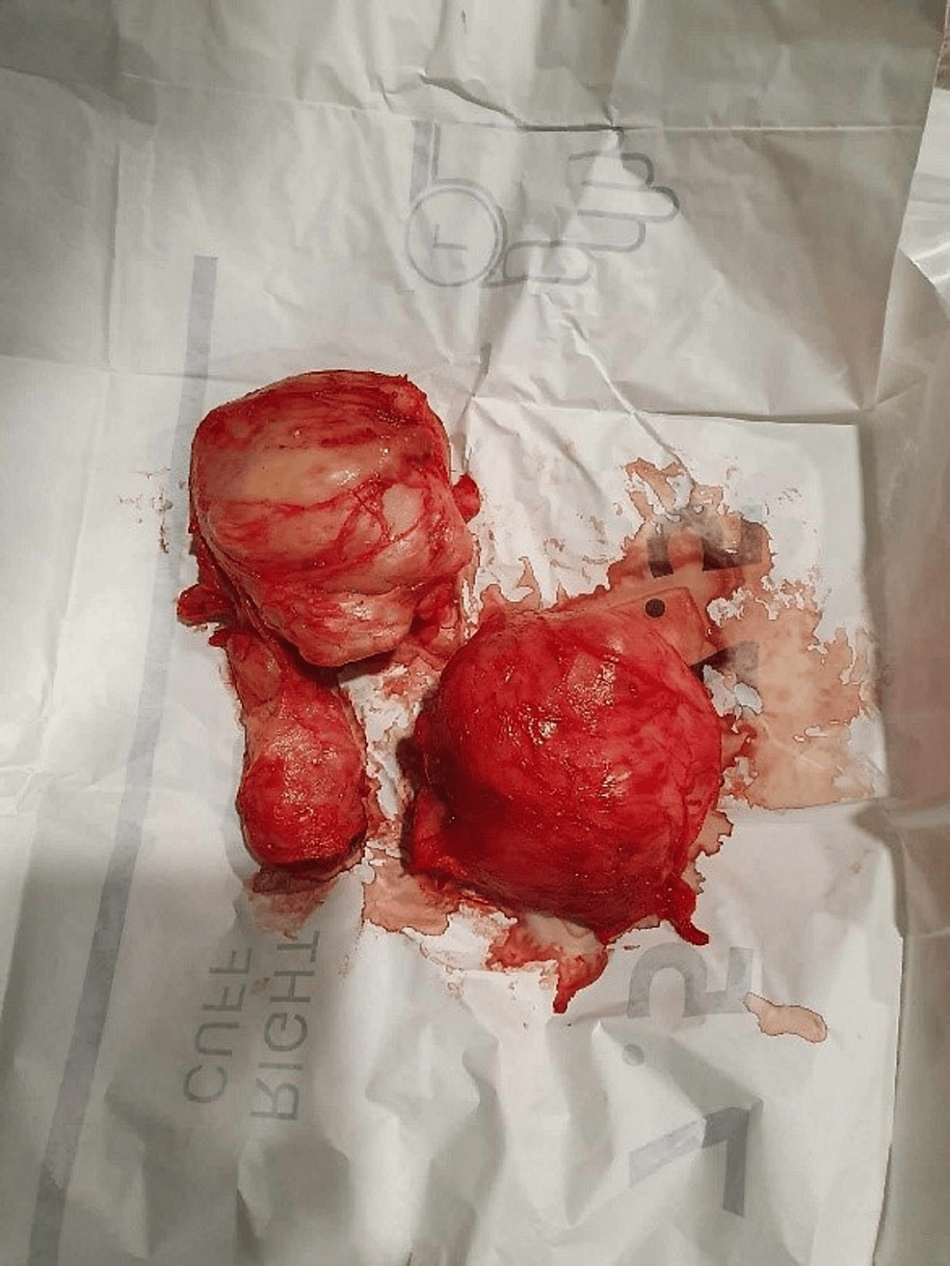
Adenomatous node removed via an intravesical approach

**Figure 2:**
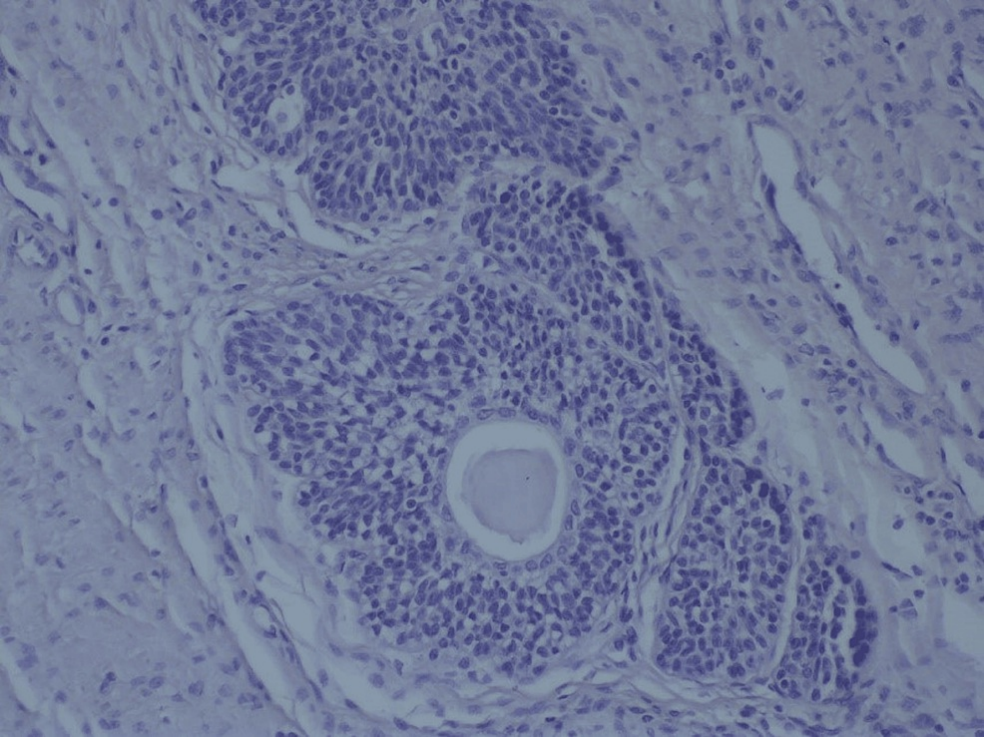
BPH (H&E, 400×) BPH, benign prostatic hyperplasia

Following the diagnostic finding of an enlarged prostate and associated voiding symptoms, the patient decided on a transvesical prostatectomy with enucleation of the adenoma. During the procedure, we dissected the plane between the prostate capsule and the adenoma, and the adenomatous node of the prostate was excised via a transvesical approach, as depicted in Figure 1. Histomorphological examination of the excised adenoma confirmed the diagnosis of BPH, consistent with preoperative assessments in Figure 2. Subsequently, an epicystostomy tube along with a urethral balloon catheter was placed to facilitate bladder drainage and ensure urinary continence postoperatively.

The patient’s postoperative course was uneventful and progressed favorably, with primary wound healing observed. The urethral catheter removal occurred on the seventh postoperative day, followed by the removal of the epicystostomy tube on the 14th day without complications. Assessments revealed encouraging outcomes: at the one-month follow-up, the patient demonstrated a residual urine volume of 104 ml, indicating a significant reduction from preoperative levels. Additionally, his IPSS had decreased to 18, indicating an improvement in urinary symptoms, while the peak urinary flow rate (Qmax) had increased to 9 ml/s, suggesting enhanced urinary flow.

With regular follow-ups, further improvements were observed. At the three-month follow-up, the residual urine volume further decreased to 10 ml, indicating enhanced bladder emptying efficiency. Moreover, the IPSS decreased to 11, reflecting a considerable improvement in urinary symptoms. The Qmax improved significantly to 13 ml/s, indicative of continued enhancement in urinary function. Importantly, the patient reported a significant improvement in his quality of life concerning urination.

According to the IPSS, the quality of life score at one month was 6, at three months it was 4, at six months it was three, and at 12 months, it was approximately 1.

## Discussion

Surgical intervention becomes necessary when the patient develops acute or chronic urinary retention, obstructive nephropathy, or severe LUTS refractory to medical treatment. BPH, BPE, and LUTS are among the most common conditions affecting aging men. Prostatic hyperplasia occurs due to the unregulated proliferation of epithelial and stromal cells, smooth muscle, impaired apoptosis, and glandular epithelium within the prostatic transition zone. Androgen receptors in BPH tissue are activated by dihydrotestosterone, synthesized by the 5α-reductase enzyme, whose activity can be countered by finasteride and dutasteride [6]. Additionally, the intrinsic impact of prostatic inflammation and metabolic parameters on the development of BPE and LUTS is increasingly recognized.

In some cases, the prostate enlarges massively, exceeding 500 g, and this condition represents an extreme manifestation of BPE called GPH. While the precise etiology of GPH is unknown, emerging evidence suggests an imbalance between growth-promoting and inhibitory factors within the prostatic gland. Mutations in specific proto-oncogenes like Ras and c-erbB-2 may disrupt the balance of signaling pathways, promoting uncontrolled signals for cellular proliferation. Additionally, alternation in p53 may contribute to the development of GPH by mutation or deletion through that gene [7].

LUTS in elderly men primarily result from urodynamic changes in the lower urinary tract, such as benign prostatic obstruction and detrusor overactivity or underactivity. These symptoms can be categorized into storage symptoms (urgency, frequency, nocturia, and urge urinary incontinence), voiding symptoms (reduced flow and feeling of incomplete emptying), and post-void dribbling.

Surgery may be required for BPH when patients encounter complications such as urinary tract retention, urinary tract infections, bladder stones, or recurring gross hematuria. These issues arise from an enlarged prostate and/or persistent LUTS despite medical interventions [8].

The surgical management of symptomatic BPH can be categorized into three main approaches: (1) minimally invasive surgical therapies (MIST); (2) simple prostatectomy; and (3) transurethral procedures.

Transurethral resection of the prostate (TURP) is widely regarded as the gold standard of treatment. However, in cases of GPH, it may prove impractical because of the size of the prostate gland, accompanied by challenging homeostatic control.

Transurethral surgery entails the extraction of obstructing adenomatous tissue through the urethra, traditionally performed using monopolar electroconductive TURP. TURP is widely regarded as the gold standard of treatment. However, cases of GPH may prove impractical because of the size of the prostate gland, accompanied by challenging homeostatic control. Over time, various alternatives, such as bipolar TURP and laser-based therapies, have been developed to enhance clinical effectiveness while minimizing perioperative bleeding and associated complications in the short and long term [9]. Open surgical intervention remains the preferred modality of treatment of choice for patients with giant BPH in our clinical settings.

When patients are suitable candidates but the physical size of the prostate poses challenges for a safe or effective transurethral approach with the surgeon’s expertise, alternative options such as simple prostatectomy (also known as adenoma enucleation) may be contemplated.

Open surgical enucleation, performed either via a suprapubic (transvesical) or retropubic approach, has traditionally been the recommended technique for GPH. More recently, laparoscopic and robotic simple prostatectomy have emerged as less invasive alternatives and are considered less invasive compared to open simple prostatectomy [10,11]. Studies have shown that minimally invasive approaches offer promising results, associated with less postoperative catheter time, a shorter hospital stay, fewer complications, and favorable results compared to open prostatectomy.

In certain cases, recent advancements in MIST offer office-based treatments that eliminate the need for regional or general anesthesia, hospitalization, cessation of anticoagulation therapy, or conventional surgery. After any prostate procedure, it is possible to experience side effects. These may vary depending on the specific procedure and can include unintended urinary incontinence, urinary tract infections, retrograde ejaculation, bleeding, and erectile dysfunction.

## Conclusions

BPH and LUTS pose significant health concerns for aging men. Surgical intervention becomes necessary for complications unresponsive to medical treatment, offering various approaches according to individual patient needs. In this research, we present the successful enucleation of a giant BPH weighing 600 g without any significant complications using a transvesical prostatectomy technique. Open simple prostatectomy remains an effective intervention for symptomatic giant BPH in our immediate community.
